# Saccadic eye movements in neurological disease: cognitive mechanisms and clinical applications

**DOI:** 10.1007/s00415-025-13275-x

**Published:** 2025-07-27

**Authors:** Yong Lin Wang, Mahima Kapoor, Joanne Fielding, Stephen Reddel, Chao Zhu, Meaghan Clough, Mina Botrous, Mastura Monif, Anneke van der Walt

**Affiliations:** 1 Department of Neurosciences, Central Clinical School, Alfred Centre, Monash University, Melbourne, VIC Australia; 2 Alfred Health, Melbourne, VIC Australia; 3 Brain and Mind Centre, University of Sydney, Sydney, NSW Australia

**Keywords:** Neuroophthalmology, Cognitive science, Saccadic eye movements, Neuropsychology, Neurological disease

## Abstract

Saccadic eye movements are rapid, precisely coordinated shifts that centre the fovea on a visual target. Their control relies on the integration of cognitive processes spanning multiple brain regions. High-resolution video-oculography enables precise measurement of saccadic dynamics, offering a window into disruptions affecting these networks. This review examines the neuroanatomy and physiology of saccadic eye movements, emphasising the cognitive mechanisms underlying their control and the methodologies used for their assessment. We synthesise evidence from saccadic eye movement testing across a spectrum of neurological diseases, highlighting its potential as an early and sensitive biomarker for detecting subclinical disease impact. While current findings underscore its promise as a non-invasive, objective tool for tracking neuropsychological dysfunction in these various diseases, we also address existing limitations and critical directions for future research towards improving clinical utility.

## The ocular motor system

Advances in eye tracking technology with high-resolution video-oculography systems, paired with cognitive tasks, have enabled detailed investigation of the “higher-level” aspects of eye movement control. This type of ocular motor testing has been seen to be sensitive to a wide range of brain pathology. The control of eye movements involves the entire brain—from the cerebral cortex to the brainstem, spanned by networks which also involve the deep grey nuclei and the cerebellum. In particular, saccadic eye movements are rapid eye movements that centre the fovea on a target and can be made in response to various sensory stimuli. They can be made reflexively towards novel visual or auditory stimuli or made to endogenously desired targets, remembered targets, in search patterns of visual scenes or even in the dark. Their successful execution depends on multiple cognitive processes involving networks widely distributed throughout the brain. When neurological disease also impacts these networks, their pathology can be reflected in disturbances to these saccadic eye movements. The detailed resolution at which these saccades can be captured enables sensitive detection of these disturbances that surpass the limits of usual clinical assessments of these functions. Impairments to saccadic eye movements, in relation to cognitive function, have been and continue to be assessed in an increasing number of neurological conditions. The current state of this research will be reviewed here as it becomes increasingly relevant to the clinician.

### Anatomy and physiology

Visual information from the retina reaches the primary visual cortex of the occipital lobe via the retino-geniculo-cortical pathway and the superior colliculus via the retinotectal pathway. From here, they are processed and integrated across several networks to eventually affect, among other things, motor control of the eyes [1]. The processing of this visual sensory information relies on cognitive processes such as attention, memory and executive function which includes reasoning and decision-making [2].

**Fig. 1:**
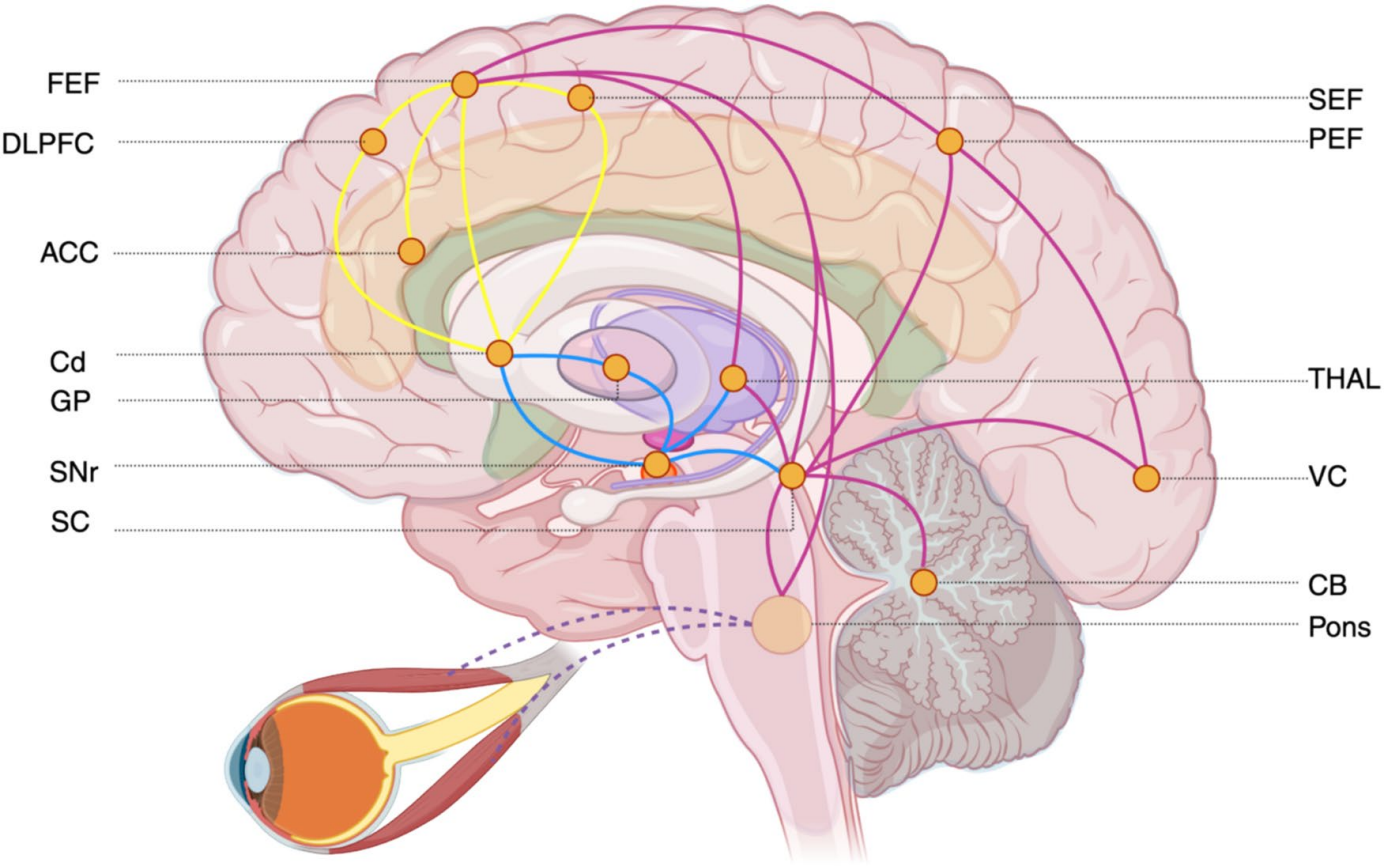
Schematic of the neural circuitry underlying saccade generation, highlighting pathways for volitional and reflexive eye movements. Volitional saccades are initiated in the frontal cortex (yellow connections) and include the frontal eye fields (**FEF**), dorsolateral prefrontal cortex (**DLPFC**), anterior cingulate cortex (**ACC**) and supplementary eye fields (**SEF**), which exert top-down control over subcortical structures. Reflexive saccades (purple connections) are driven by bottom-up visual input from the visual cortex (**VC**) projecting to both the parietal eye fields (**PEF**) and FEF, thalamus (**THAL**) and basal ganglia. Both cortical streams influence the basal ganglia network (blue connections) at the caudate nucleus (**Cd**), which modulates output via the direct and indirect pathways through the globus pallidus (**GP**) and substantia nigra pars reticulata (**SNr**). The cerebellum (**CB**) and thalamus provide additional modulator input. Both volitional and reflexive pathways converge on the superior colliculus (**SC**) and downstream brainstem premotor circuits (**pons**) to execute saccades. Created in BioRender. Wang, YL. (2025)

Saccades are conjugate, ballistic eye movements that can reach speeds of up to 700 degrees per second, the purpose of which is to centre a visual target on the fovea, which is a small area of retina with the greatest cone photoreceptor density responsible for central visual acuity. This allows for high-acuity “seeing” of the target [3]. Repeated foveations are required to obtain the necessary, desired, or goal-relevant visual information from any scene. This is achieved by saccadic eye movements that can occur several times each second [4]. The decisions, conscious or not, about which targets to look at and the subsequent execution of saccades towards these targets are the outcomes of cognitive processes. The processes underlying saccades can be delineated in ocular motor studies, with particular emphasis on differentiating reflexive from volitional (or endogenous) saccades. While looking at a newly appearing visual target can seem reflexive (and mechanisms are described to facilitate such reflexive saccades), the interplay of attention and endogenous desires complicates such circuitry. The cognitive processes underlying saccadic eye movements have been and continue to be elaborated in physiologic and pathologic states, which will be reviewed below, and summarised in Fig. 1.

The six extraocular muscles that ultimately move each eye are innervated by three motor nerves that arise from the brainstem—the oculomotor, trochlear and abducens nerves. The velocities required of saccadic movements demand powerful phasic muscle contractions resulting from a burst of action potentials called pulse innervation. At the same time, the subsequent maintenance of the eye position against viscoelastic forces that would otherwise restore the eye’s primary position requires ongoing tonic contraction. The paramedian pontine reticular formation (PPRF) and the rostral interstitial nucleus of the medial longitudinal fasciculus (riMLF) are the centres from which the saccadic pulse originates to generate, respectively, horizontal and vertical saccades [5].

The superior colliculus (SC), located on the dorsal surface of the midbrain, receives sensory information directly from the retina via neurons of the retinotectal pathway [2]. The SC acts as the main saccadic relay at the level of the brainstem, giving rise to projections towards the pontine saccadic control unit. The arrangement of the neurons in the superficial SC topographically maps the visual space, and it is thought that this representation alone can form a reflexive circuit allowing for detection of a target and generation of a saccade towards it [2]. The main outputs of the SC inhibit the tonic omnipause neurons of the PPRF and riMLF, which subsequently facilitates the “release” of a saccade by way of motor output [6]. Their counterpart, the fixation neurons, projects excitatory signals from the rostro-lateral SC to the omnipause neurons, resulting in inhibition of saccadic pulses [7].

This brainstem circuitry constitutes the lowest levels at which saccadic parameters—timing, amplitude, velocity and the maintenance of gaze—are encoded. A constant interplay of input from the saccade neurons and the fixation neurons from the SC maintain the dance of repeated foveations and sustained gaze. Further excitatory and inhibitory influences from higher centres and their balance modulate this system so that our eyes can successfully respond to the world of stimuli. The immediate inhibitor of the SC not only descends from the substantia nigra pars reticulata (SNr) and the globus pallidus internus (GPi) in the basal ganglia [8] but also receives inputs directly from the thalamus and frontal eye fields (FEF). The basal ganglia functions as a relay to integrate information from several cortical networks via the caudate nucleus [9]. The output from the SNr is predominantly strong tonic inhibition to the SC and thalamus [9, 10], which is modulated by the direct and indirect basal ganglia pathways to effect initiation, maintenance and cessation of saccadic eye movements. The thalamus further mediates feedback to the FEF [1].

The FEF, located at the lateral precentral gyrus, and the parietal eye fields (PEF), located around the intraparietal sulcus, both provide excitatory input to the brainstem. The FEF is part of the premotor cortex and has highly ramified connections with the prefrontal cortex. These connections integrate higher cognitive demands that result in output to the brainstem to generate both volitional and reflexive saccades, both directly and via the basal ganglia [11]. Lesioning studies of both the FEF and the SC have demonstrated resultant deficit to contralateral saccades, which are most pronounced and permanent only when both structures are removed [12]. This suggests that parallel to the SC projections, the FEF also projects directly to pontine centres and electrophysiological evidence has found that FEF stimulation results in inhibition of omnipause neurons of the raphe nucleus in the leadup to saccade execution [13].

The PEF serves an important node in integrating sensory and motor information. Visual input into the PEF derives from the occipital extrastriate visual cortex regions V2 and V3, which receive retinal sensory input via the lateral geniculate nucleus (LGN) [2]. The PEF then integrates this sensory information and transforms retinotopic visual stimuli into saccadic motor plans, which can be held in working memory [11]. The PEF plays a key role in the generation of reflexive saccades, further modulated by its function in visual attention and selection of salient stimuli [14].

The supplementary eye fields (SEF), located at the superior parietal sulcus, support the generation of voluntary saccades and are extensively connected to all other cortical regions involved in eye movement control, including the FEF and the dorsolateral prefrontal cortex (DLPFC). Their role in saccadic eye movements is in both temporal and spatial coordination, especially in relation to bodily movement [15].

The DLPFC plays an important executive function in decisional processes. In relation to saccadic eye movements—which often reflect outcomes of decisions between competing demands—it prepares saccades in anticipation of release and can modulate reflexive saccades [15]. The latter function sees the integration of volitional impulses from the FEF or SEF to inhibit reflexive saccades generated from the PEF. In conjunction with stopping unwanted reflexive saccades, the DLPFC also prepares subsequent volitional saccades and has been shown to play an essential role in saccadic tasks involving spatial memory and prediction (for example, anticipating the location of a moving target) [15].

Further modulation of saccades occurs with input from the ventral paraflocculus (VPF) and the fastigial oculomotor region (FOR) of the cerebellum, which are responsible for refining saccadic accuracy [16]. Projections from the SC to the cerebellum via the basal ganglia allow the cerebellum to monitor and modulate saccadic efferents by way of output to the burst generator of the SC. Impairments here result in reduced accuracy and increased variability in saccadic amplitudes [17].

Overall, the separation of saccades into those that are reflexive and those that are volitional is a conceptually useful approach, which is reflected in Fig. 1, especially in view of how they are assessed in the ocular motor tasks described below. However, these neuroanatomical bounds are far from clear and their degree of separation is contentious. Reflexive saccades are bottom-up responses to environmental stimulus, and the final saccadic output relies on connection between both the PEF and the visual cortex to the SC. Volitional saccades are top-down, internally driven responses involving goals and desires and depend on more reciprocated networks between the FEF, SEF and the DLPFC with eventual output to the SC mediated by the basal ganglia [3].

### Saccadic eye movements and their assessment

With recording and measurements obtained by means of video-oculography, multiple testing paradigms can be used to explore saccadic eye movements and their control. In video-oculography, gaze position is determined from continuous video monitoring of the eyes where various features of the eye, and sometimes the surrounding orbital structures, are used to estimate the direction of gaze [18]. For example, in infrared video-oculography, eye features—in particular, the pupil, its centre and corneal reflection from a fixed infrared light source—are calibrated within a fixed geometry which includes the camera, the screen with visual targets as well as the light source. The movements of the pupil are tracked as well as changes to its relation with the other features, and together these form anchor points from which to calculate the direction in which the eye is looking. The temporal and spatial accuracy is improved in various iterations of this technology by using cameras with increased resolution and sampling rate, and by tracking additional features such as eye corners or scleral landmarks. Thus, the coordinates of gaze and its change over time are measured precisely and used to determine parameters such as the beginning and end of a saccade, the acceleration and velocity of the eye movement and its overall accuracy in relation to various test targets, which will be explored below.

The tasks that constitute the ocular motor tests are presented on a computer monitor and are generally simple and brief. Stimuli appear on the screen and instructions are provided about what to look at and when. This allows, after initial explanation and coaching, well-tolerated repetition of many test trials to rapidly obtain accurate and reproducible results. Several benefits are apparent—the simplicity of the tests reduces the impact of learning effect, and the tests can proceed rapidly without implicit feedback, mitigating the impacts of performance-related anxiety that can often confound standard neuropsychological testing.

### The prosaccade task

**Fig. 2:**
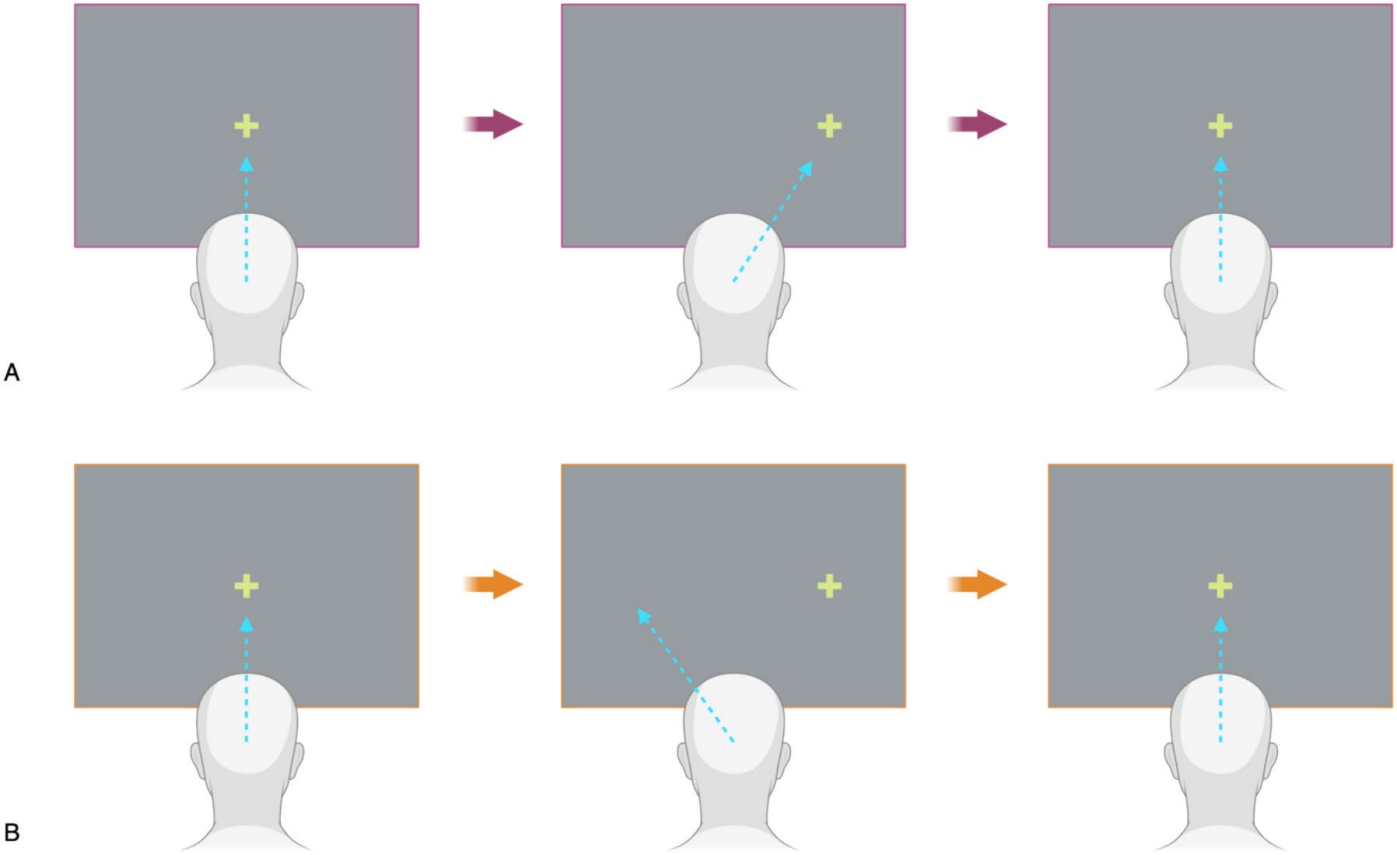
Temporal and spatial schematics of A, the prosaccade task and B, the antisaccade task. In the prosaccade task, the participant is instructed to saccade to a visual target that appears randomly either to the left or the right quickly and accurately. In the antisaccade task, the participant is asked to saccade to an imaginary, mirrored location of the appearing target. Created in BioRender. Wang, YL. (2025)

The prosaccade (PS) task is also described as a visually guided task and requires the participant to look at a central fixation target and then saccade to a newly appearing visual target, generally towards the left or right of the fixation point, quickly and accurately (Fig. 2A). Latency to initiation of the saccade, its acceleration, velocity and accuracy can all be measured. Conceptually, this task represents interrogation of the reflexive ocular motor circuit, which although “reflexive” still demands some amount of cognitive processing. Its successful execution depends on the interplay between spatial attention (noticing when and where a new target appears) and saccade planning (how to move the eyes to foveate the new target), both of which demand cortical inputs. There is ongoing debate regarding the nature and degree of such interplay; for example, Hunt and Kingston argue that separate neural systems are responsible for spatial attention and direct visual attention [19]. Since one can covertly direct attention towards something in the peripheral vision without necessarily executing an overt saccade towards it—these “covert” versus “overt” shifts thus might represent neuroanatomical separation. Conversely, Rizzolatti et al. argue that such covert shifts already entail saccade planning, and in this view, saccades are necessarily entangled with attention [20]. Of note, covert shifts are inherently part of the antisaccade task (see below), requiring participants to both pay attention to peripherally appearing targets while also inhibiting a reflexive saccade. Functional neuroimaging studies have demonstrated that both covert and overt shifts involve activity in the cortical ocular motor control areas—specifically the precentral sulcus (corresponding to the FEF), the interparietal sulcus (corresponding to the PEF) and the lateral occipital cortex. However, visual attention that results in release of an actual saccade correlates with greater intensity of activity in these areas [21].

Furthermore, the measurement of PS latency can be complicated by the so-called gap and overlap effects. The gap effect emerges when the onset of the new visual target is temporally delayed relative to the disappearance of the central fixation point. A gap—for example, of 200 ms—between the disappearance of the fixation point and the appearance of the target has been shown to reduce latency. The opposite of the gap effect, the overlap effect, occurs when the fixation point persists and temporally overlaps the appearing target, resulting in prolonged latency [22]. Two main hypotheses have been proposed to explain these effects. The first hypothesis is that a gap allows for the disengagement of attention [23] mediated by a disruption in various attention and fixation mechanisms [24]. Conversely, the overlap effect is seen by preventing disengagement. The second hypothesis is that the gap acts as a warning mechanism to trigger motor system preparation [25].

In some trials, the gap effect facilitates the occurrence of an “express” saccade that occurs at around 70–110 ms compared to the standard prosaccadic latency at 200 ms, resulting in a bimodal distribution of saccade onset [26]. These express saccades are thought to represent more reflexive output generated entirely from lower-level saccade preparation [27]. However, experiments have shown that they, too, can be influenced by higher-level instructions such as those in a specific visuomotor task set [28], where verbal instructions that set up a particular sensorimotor expectation (the visuomotor set) have been demonstrated to influence both express saccades and saccades of the usual latency. This suggests that some degree of cognitive input modulates even the more reflexive aspects of the ocular motor system.

### The antisaccade task

The interplay between reflexive saccades and their inhibition can be further explored in ocular motor testing by the antisaccade (AS) task. This task requires participants to fixate on a central point, after which a new target appears on either its left or right. Instead of looking at the new target (as in the PS task), the participant is asked to saccade to its mirror opposite location—an imaginary location—in relation to the original central fixation point (Fig. 2B). Conceptually, this task attempts to isolate the volitional aspect of saccadic control and, when broken down, requires several component steps of processing beyond the simpler PS task. Initially, the prepotent reflex towards the actually appearing target requires suppression; in other words, the PS needs to be inhibited. Subsequently, an interplay of other cognitive processes generates the resultant volitional saccade to the imaginary, endogenously desired target. If the prepotent reflex is not suppressed, an error is seen as the eye saccades towards the incorrect target. Whether or not these errors are self-monitored and corrected depends on prefrontal and anterior cingulate cortical (ACC) function [29].

Suppression of the reflexive PS at the SC, in the first order, requires inhibitory signal from the DLPFC. The DLPFC has a prominent role in many executive functions, including behavioural choice, and here functions to resolve conflicting decision-making rules [30]. In preparing saccades, its role is crucial in integrating competing saccadic demands [31]. Here, there is also a contribution from the ACC, where impairment has been associated with increased error rates [32].

After this, planning and executing the opposite saccade requires input from the posterior parietal cortex for the vector inversion and the FEF to initiate the resultant AS [33]. These extra steps of the process require more time, which manifests as greater latency between the onset of the cue and the commencement of the saccade compared to the PS task [22]. Interruptions along this extensive network can add further latency delays or cause increased errors altogether.

Neuroimaging studies using functional MRI and FDG-PET have been crucial to clarify many of these anatomical correlates. Antisaccadic tasks correlate with increased activity of the frontoparietal networks that participate in the generation of both PS and AS [34]. These tasks also place increased demands on the DLPFC [35]. Thus, the AS task needs multiple cognitive processes to dovetail—from attention, working memory, inhibition and decision-making—in order to generate a correct saccade. With video-oculography, the parameters of this task can be precisely measured to reveal problems in this system.

### The switch cost

In the AS task, the inhibition of the reflexive saccade has been shown to have lingering effects on subsequent PS when these tasks are interleaved—that is, when a person is asked to perform a PS task immediately after being asked to perform an AS task in consecutive trials. This so-called switch cost has been demonstrated in several studies and is thought to be mediated by a residual inhibitory response state after an AS is performed. This ultimately results in increased latency when there is a subsequent switch to a PS task [36]. This switch cost is generally considered unidirectional in that a PS task preceding an AS task does not influence the latency of the latter. Interleaving the PS and AS tasks provides further opportunity to detect impairments in cognitive control, where increased susceptibility to task switching might reflect impairments to working memory and response inhibition [37].

### Memory-guided saccades

Memory-guided saccades constitute another set of endogenous saccadic paradigms that calls on the participant to look at the remembered position of a visual target that has already disappeared, with some variable period of delay after its disappearance before the saccade is cued. Its simplest iteration asks participants to fixate on a central target while a peripheral target is briefly displayed. Sometime after the peripheral target disappears, a cue is given to the participant to saccade to where the peripheral target was. Successful execution has several cognitive demands. Both FEF and PEF, which both have established roles in PS execution, participate in both the initiation and accuracy of memory-guided saccades. The prefrontal cortex plays a vital role in spatial memorisation and attention where, once again, the DLPFC is required to suppress reflexive saccade towards the initially appearing target [38]. The additional processing to achieve this involves coordination of widespread connections, and as a result, memory-guided saccades are seen to have increased latencies compared to PS [39]. Increasing delay to cue the saccade to the remembered target can shift cognitive demand between short-term or working spatial memory, to long-term memory [40]. Variations of memory-guided testing exist, for example, by using an *n-*back task that requires the participant to saccade towards the correct *n*-back location with a cue that specifies the *n*. This further loads working memory to heighten sensitivity to disturbances in this system [41].

Overall, the temporal and spatial resolution of video-oculography systems can detect subtle alterations in ocular motor outputs that require coordination of multiple brain networks. Furthermore, novel testing paradigms can be constructed, or existing paradigms can be further altered to allow for granular control over precisely what aspects of cognition are being tested. This adaptability allows assessments to be maximally exacting of the proposed cognitive impact of any disease. Well-designed testing paradigms in conjunction with accurate normative data imbue ocular motor studies great potential as a paraclinical test in neurology and neuropsychology, with exemplary findings summarised in Table 1.

**Table 1:** Ocular motor findings in different disease states

Disease	Saccadic Assessments	Psychometric Correlates	Disease Correlates	References
Alzheimer’s disease	Increased latency in PS and AS, increased error rates in AS, in both AD and MCI patients. Prominent gap effect in AD patients but not MCI	Increased AS latency and error rates correlate with worsening Stroop test and digit span	Increased AS latency and error rates correlate with decreased MMSE	[42, 43]
Parkinson’s disease	AS error rate increased in switch task. Latencies confounded by bradykinesia	Poor performance on switch task correlates to performance on assessments of cognitive flexibility in early PD	No studies correlating saccadic performance to disease attributes	[44–47]
Multiple Sclerosis	Increased error rates in PS, AS and memory-guided tasks. Increased latencies in PS and memory-guided tasks. Increased error rates in n-back task	Increased AS error rates and latencies correlate with worse PASAT performance only in late MS group	Latency increases correlated linearly with disease duration. No EDSS correlation	[48–50]
Stroke	Increased error rates in both AS and memory-guided tasks in patients with acute mild stroke	No changes in usual stroke clinical assessments including NIHSS and mRS	Improvement in OM parameters 3 months from acute stroke	[51]
Concussion	Increased AS error rates	Normal psychometric performance	Normal neuroimaging	[52–56]
Epilepsy	Longer latency, poorer spatial accuracy and increased error rates with AS task in patients with drug-resistant focal epilepsy	Reduced WAIS-IQ as predicted by NART	TLE and left hemispheric epilepsy correlated with increased AS latency. Right hemispheric epilepsy correlated with reduced AS spatial accuracy. FLE correlated with highest AS error rates	[57]
Schizophrenia	Increased AS error rates and latencies. AS error rates are also increased in patients with first episode of schizophreniform psychosis	Reduced spatial working memory performance. Correlation seen with attentional focus and inhibitory control deficits	Reduced frontal lobe volume. Little to no correlation with clinical symptoms	[58–60]
Hepatic encephalopathy	Increased AS latency and error rates seen in patients with mild hepatic encephalopathy	Increased AS latency and error rates correlate with worse performance on Digit Symbol Modalities Test, Number Connection Test and Stroop task	Increased AS latency and error rates correlate with worse encephalopathy measured on the PHES scale	[61]
Cervical dystonia	Increased AS latency and error rates. Increased express saccades	No studies correlating saccadic performance to neuropsychological assessment	No studies correlating saccadic performance to disease attributes	[62]

*AD Alzheimer’s disease, MCI mild cognitive impairment, AS antisaccade, PS prosaccade, NIHSS National Institute of Health Stroke Scale, mRS modified Rankin scale, MMSE mini-mental state examination, PASAT paced auditory serial addition test, EDSS expanded disability status scale, OM ocular motor, TLE temporal lobe epilepsy, FLE frontal lobe epilepsy, WAIS Wechsler Adult Intelligence Scale, NART National Adult Reading Test*

## Ocular motor outcomes in neurological disease

### Overview

Diefendorf and Dodge first measured eye movement abnormalities in 1908 by asking psychiatric patients to follow a swinging pendulum, and during this pursuit, light reflected from the cornea would expose a falling photographic plate, leaving a time-varying trace of eye position with millisecond resolution [63]. They found prolonged reaction times and various disturbances to the pattern of pursuit across patients in differing states of psychoses. Up to now, the largest body of work concerning eye movement abnormalities remain within schizophrenia, alongside work in cognitive impairment and especially Alzheimer’s dementia—both diseases being leading causes of disease burden worldwide. With increased availability of eye tracking devices alongside evolution in practicality and precision, these assessments have now been undertaken in an expanding number of neurological conditions, as summarised in Table 1. These applications are reviewed below, with an emphasis on subclinical findings that help extend typical clinical assessments.

### Schizophrenia and mental health disorders

The pathophysiology of schizophrenia is reflected in findings of reduced grey matter volume in the frontal and temporal lobes, with frontal lobe hypometabolism being a well-demonstrated neuroimaging correlate [64]. Ocular motor studies demonstrates consistently impaired AS performance in schizophrenia, with findings of increased error rates and latencies replicated across dozens of studies [58], the earliest published by Fukushima et al. in 1988 [65].

Hutton et al. demonstrated increased error rates in the AS task in 109 patients with first presentation of schizophreniform psychosis [66]. This correlated with impaired working memory on neuropsychological assessments [59]. These findings suggest that impaired working memory rather than inhibitory failure drives error rates in this population. The presence of these changes in patients presenting with first episode of psychosis is of special interest given that these presentations occur long before the more chronic complications of schizophrenia or its medical treatment introduce confounders. Further support comes from a neuroimaging study in first-episode psychosis. The authors demonstrated ocular motor impairment, specifically AS errors, correlated with reduced premotor cortex volume [60]. These alterations to saccadic parameters, in association with both neuropsychological and neuroimaging changes, point towards DLPFC dysfunction in schizophrenia. Together, these lend weight to the utility of saccadic eye movement disturbance as a biomarker in schizophrenia, with attempts seen to combine saccadic parameters into diagnostic scores [67].

Beyond schizophrenia, depression and anxiety are well-recognised cognitive confounders in standard neuropsychological testing, which have also been found to influence cognitive control of saccades. Compared with healthy controls, longer latencies and increased error rates have been reported in assessment of AS in depression [68, 69], alongside reduced accuracy in memory-guided tasks [68]. In anxiety, AS latencies were found to be increased but not error rates, and PS parameters were unaffected [70]. While the overall volume of research remains small, given the prevalence of comorbid anxiety and depression in many neurological diseases, their effects on saccadic parameters must be accounted for in any study methodology.

### Alzheimer’s disease

Alzheimer’s disease (AD) is underpinned by progressive neurodegeneration mediated by the accumulation of amyloid beta peptide as well as tau protein, which together result in neurotoxicity [71]. While memory impairment and hippocampal atrophy are the most characteristic regional findings in AD, neurodegeneration affects the brain as a whole and generalised atrophy is frequently seen, especially later on in the disease. This is reflected in impairments of other cognitive domains, including visuospatial and executive function. This is well characterised by FDG-PET, which can demonstrate hypometabolism beyond the hippocampi and involves areas in the frontal, parietal, posterior-temporal lobes and basal ganglia [72]. As such, it is not surprising that the ocular motor system is affected, even early on in its disease course, such as in mild cognitive impairment (MCI), which is considered a transitional or prodromal phase of cognitive decline that is yet to declare itself as clinically definite dementia.

In the PS paradigm, latency, velocity and accuracy have all been shown to be impaired in AD (reviewed by Molitor et al. [42], Wolf et al. [73] and Opwonya et al. [43]). Latency has been the most consistently impaired parameter, while impairment of velocity and accuracy is demonstrated with less consistency between studies. Yang et al. demonstrated prolonged latencies in people with both amnestic MCI (aMCI) as well as mild to moderate AD [74]. In addition there was a correlation between increasing latency and reduction in mini-mental state examination (MMSE) score within the AD cohort [74]. Of note was methodological attention paid to gap and overlap conditions, where the gap effect was found to be exaggerated in the AD cohort compared to the control group. While the presence of a gap effect is expected in a normal cohort, that is, saccadic latency is lower in gap trials compared to overlap trials, they found that this difference is larger in the AD cohort. This suggests that the disengagement of attention, by using a gap, plays an outsized effect on saccade initiation in AD patients. This is clinically important as it supports the importance of visual attention in the landscape of AD impairments, where impairment to attention is an early disease feature [75].

In AS trials, both latencies and error are increased in AD patients compared to normal controls and can be correlated with neuropsychological testing scores, including MMSE, Stroop testing and digit span [42, 76]. This points to deficits in inhibitory control and working memory, both involving the DLPFC [77]. Furthermore, AD patients demonstrate less capacity to correct erroneous saccades [43], reiterating inhibition and error monitoring impairments. These changes have also been found in patients with amnestic MCI [78, 79]. However, it remains unclear if saccadic parameters can differentiate between types of MCI in order to anticipate progression towards AD reliably [73].

In the meta-analysis by Opwonya et al. [43], most of the included studies demonstrated longer latencies in both PS and AS paradigms. Further increased AS error rates and decreased amplitudes (that is, hypometric saccades) were common in cognitively impaired patients. Of note from this meta-analysis is the finding of a significant difference between MCI and AD patients, where the gap effect was greater in the latter population under PS conditions. Although this finding is derived from a limited number of studies, it might indicate that the degree of cognitive impairment can be correlated to performance in ocular motor testing paradigms that demand increased attention. Once again, methodological heterogeneity was noted between the included studies, where the significance of the gap-overlap effect on latencies are overlooked in some studies. As discussed earlier, if these effects are not carefully attended to in the study design, real differences between populations may be masked.

In summary, the existing literature demonstrates that ocular motor testing can help clarify the disease processes of AD and the affected brain networks. The correlation of ocular motor testing with cognitive testing outcomes points towards its potential utility as a biomarker, especially during the long prodromal phase of the disease before obvious clinical symptoms are manifest.

### Parkinson’s disease

Impairment of saccadic eye movements is likewise seen in Parkinson’s disease (PD). PD is another neurodegenerative disease where the accumulation of alpha-synuclein results in the failure of dopaminergic systems and can lead to dementia [80]. The movement disorder that accompanies the loss of dopaminergic neurons of the midbrain often causes hypometric saccades and is already a well-recognised ocular feature of the disease.

Suspicion for early PD is primarily based on the presence of motor symptoms—that is, bradykinesia, rigidity and tremor—and cognitive impairment is generally not a clinical issue at this stage. However, saccadic eye movements assessed in 19 drug-naïve early PD patients found that while PS parameters were similar to healthy controls, the complication of the switch task resulted in a significantly higher AS error rate among the PD group [44]. This was also correlated with poorer performance on the Birmingham Rule Finding and Switching test, which assesses cognitive flexibility that similarly makes demands of prefrontal cortex. The AS error rate remains a marker of cognitive function in this setting, while the comparison of inter-individual latencies could be confounded by dampening introduced by bradykinesia [45]. In fact, latency results across studies in PD have demonstrated a large amount of heterogeneity, even when assessing only the reflexive PS [46]. Other studies convincingly demonstrate impaired AS latency, error rates and higher error rates in memory-guided tasks among PD patients [47]. A finding of an increased proportion of express saccades in the PS task has also been made in PD [81], where problems inhibiting saccades may correlate to larger problems with supressing automatic responses seen in the disease [82]. Overall, these findings suggest impairment of inhibitory control and working memory executive function that is consistent with dopaminergic impairment along the prefrontal-striatal circuitry described in PD [83]. Additional contribution to these impairments is seen in advanced disease from cortical Lewy body deposition involving the ACC and the entorhinal cortex [84].

The saccadic eye parameters changes seen in both the AD and PD literature, especially in early disease, are predominantly from cross-sectional populations. Longitudinal follow-up with ocular motor assessments remains essential to properly delineate saccadic changes over the disease course. Changes over time must be correlated to early associations with disease phenotypes to establish a robust biomarker. This is an especially important task given the potential utility of saccadic eye movement assessment to monitor treatment response in clinical trials, especially for novel AD therapies where efficacy will likely be in early disease.

### Multiple sclerosis

Multiple sclerosis (MS) is an autoimmune neurological disorder whose spectrum of disease likely also encompasses a neurodegenerative component beyond the discrete, focal relapses characteristic of typical MS attacks in relapsing–remitting disease [85]. The Fielding group demonstrated impaired saccadic eye movements in MS that correlates with cognitive impairments, specifically attention and working memory [86]. In memory-guided saccades, latencies and errors were increased in MS patients compared to controls. This suggests impairment of working memory and inhibitory control, which correlated with performance on corresponding neuropsychological assessments [86]. Here, the Paced Auditory Serial Addition Task (PASAT), the California Verbal Learning Test (CVLT), the Symbol Digit Modalities Test (SDMT) and the backward digit span were used to assess attention, working memory and processing speed. In further work, inhibitory control deficits, as reflected in increased error rates in AS and memory-guided tasks, were found in MS patients compared to control [49]. Latencies in the PS and memory-guided tasks were found to increase linearly with disease duration [49]. An experiment using an ocular motor n-back task revealed increased error rates suggesting reduced working memory capacity. This was again linearly associated with longer disease durations [50].

Most importantly, changes in ocular motor tests were observed in people with MS without any corresponding changes in neurological function. Specifically, the Expanded Disability Status Scale (EDSS), a widely applied scale to quantify disability progression in MS, remained stable despite increasing antisaccade error rates and latencies in MS patients over two years [48]. While not a saccadic eye movement task, a measurement of smooth pursuit accuracy and closed-loop pursuit gain was reduced even in patients with clinically isolated syndrome (CIS) [87], the earliest clinical manifestation of MS. In another study with an endogenously cued saccade paradigm that assesses inhibitory control, not only were significant deficits found in MS patients in general but also in MS patients without any other clinical evidence of disability (that is, they had an EDSS score of 0 and normal performance on neuropsychology assessment) [86]. This demonstrates that ocular motor assessment has the potential sensitivity to resolve granular pathology beyond what is feasible in routine clinical practice.

### Stroke

Ischaemic stroke occurs most commonly secondary to vasculopathy, resulting in impaired blood supply and focal brain infarction. Cognitive impairment can result from one stroke, depending on its size and location, or from the accumulation of many strokes. In addition, “covert” small vessel disease results in more diffuse brain injury that can ultimately lead to vascular dementia [88]. It is noteworthy that many of the original studies elaborating cortical ocular motor control relied on patients who were found to have small ischaemic strokes affecting the area of interest. For example, Rivaud et al. [89] described eye movement abnormalities in patients who had ischaemic stroke in the frontal eye field, while Pierrot-Deseilligny et al. [31] assessed patients with ischaemic stroke in the DLPFC to further delineate its role in ocular motor behaviour. More generally, the effects of stroke on ocular motor performance was evaluated in 15 patients with mild stroke (National Institute of Health Stroke Score, NIHSS ≤ 6) without visual defects or gaze palsy when compared to control [51]. Stroke locations among these patients were distributed between cortical and subcortical locations, with the majority found in the frontal, temporal and parietal lobes. In both AS and memory-guided saccade tasks, stroke patients made more errors than the control group. The authors demonstrated quantitative improvement in these same measures over a period of 3 months from initial stroke onset. Usual bedside clinical scores obtained alongside ocular motor testing such as NIHSS, modified Rankin score (mRS) or the mini-mental state examination (MMSE), were normal. These results again highlight the existence of subclinical cognitive dysfunction to which these ocular motor assessment methodologies are especially sensitive. More intensive neuropsychological testing may have identified corresponding deficits but was not performed as part of this study. These neuropsychological assessments are time consuming and not routinely performed in clinical practice following strokes of mild severity. The use of ocular motor tests that can detect subtle deficits with high sensitivity represents a plausible and practical approach.

### Concussion

Concussion or mild traumatic brain injury (mTBI) results from acceleration–deceleration forces applied to the head. This leads to brain injury via several mechanisms, including focal lesions such as contusion and more diffuse injury through widespread disruption of axonal function of varying severity [90]. Cognitive impairment can occur eventually, especially with recurrent episodes of head trauma culminating in, for example, chronic traumatic encephalopathy seen in sports-related concussion (SRC) [53]. Several studies have found increased saccadic latencies and errors in people with both TBI or mTBI compared with controls, which are variably reflected in comparative neuropsychology assessments (for example, see Kraus et al. and Ting et al. [54, 55]).

Clough et al. found evidence of ocular motor testing abnormalities in 15 Australian rules footballers with a history of SRC when compared with athletes who had no history of head trauma [52]. The study population consisted of young athletes with a mean age of 24 years and low exposure to SRC. The footballers were asymptomatic of the concussive event and assessed well outside of the acute post-concussive period—6 months or longer after the initial injury. Testing included a switch task where PS and AS tasks are interleaved. This allowed for investigation of inhibitory control and performance under cognitive load. Footballers demonstrated shorter PS latencies and increased AS error rates compared to controls, suggesting impaired inhibitory control.

The ocular motor findings occurred without the advent of any clinically discernible brain injury or cognitive impairment. Concurrent MRI brain, including advanced functional imaging and diffusion tractography, did not reveal significant structural or functional differences between the football players and controls. These abnormalities were present over 6 months since the last known concussion and support persistent pathological cognitive or neuronal changes due to mTBI that can only be detected using sensitive saccadic eye testing paradigms.

Other studies have also demonstrated a dose effect, with a correlation between the number of sports-related head impacts and worsening saccadic latency and error rates [56]. Participants of Canadian college football, another contact sport, were followed for one to two seasons with a helmet-mounted accelerometer used to count head impacts. The athletes were assessed with PS and AS batteries on four occasions from baseline and throughout the season. The authors reported linear correlations between the cumulative number of head impacts and increased PS and AS latencies, but not error rates. These findings are clinically impactful and demonstrate ocular motor changes, potentially reflective of impaired executive function, after only one to two seasons of football. Notably, most of the head impact events counted in this study do not meet diagnostic criteria for concussion or mTBI. This suggests that even the accumulation of small impacts over a short duration of time could lead to neuro-cognitive pathology.

Overall, the landscape of concussion offers significant opportunities for applying saccadic eye movement assessment. The capability of detecting ocular motor disturbances before the advent of more symptomatic chronic traumatic encephalopathy would facilitate public health intervention in a preventable condition.

### Epilepsy

In focal epilepsy, there is an “enduring predisposition to epileptic seizures”, required as part of the International League Against Epilepsy’s definition of epilepsy [91], which is presumed to be due to structural or functional disruption in the brain. Temporal lobe epilepsy, most often seen with associated hippocampal sclerosis, remains archetypal for focal epilepsies due to its relative prevalence. Still, the location of seizure foci can be varied, and the causes can be wide ranging. Cognitive impairment in epilepsy is common and thought to be multifactorial, with contributions from structural disruption and ictal–interictal dysfunction. The adverse effects of antiseizure medications contribute further to cognitive dysfunction [92]. It is not surprising then that ocular motor dysfunction was found among 51 patients with focal, drug-resistant epilepsy. These patients demonstrated increased AS latency and error rates and overall poorer accuracy compared to control [57]. In a subgroup analysis, the authors demonstrated that only the temporal lobe and left hemispheric epilepsies contributed to prolonged AS latency. Conversely, patients with right hemispheric epilepsies demonstrated larger magnitudes of AS inaccuracy. These changes reflect increased processing speeds related to left hemispheric dysfunction and the right hemisphere’s dominant role in spatial attention. Seen within the context of focal epilepsy, ocular motor testing and saccadic parameters therefore have the potential to localise lesions. Of note is the absence of ocular motor tasks from this study that make specific demands on memory. Impaired verbal memory is an established finding in epilepsy, especially in association with the hippocampal atrophy seen in TLE [93]. Furthermore, the cognitive adverse effects of antiseizure medications were not quantified.

Outside of tertiary centres with comprehensive epilepsy programmes, neuropsychological evaluation of patients with epilepsy in general is not routine. Cognitive profiles in various epileptic syndromes and epileptogenic localisations continue to be described [93], and ocular motor testing potentially offers a more accessible modality to both identify issues with cognition and contribute to the localisation of epilepsies.

### Toxic-metabolic encephalopathy

Acute toxic-metabolic encephalopathy is a common and generally reversible cause of confusion and cognitive change. Here, brain neuronal activity is globally disrupted by perturbations to its local environment—be they related to energy metabolism, osmolality, temperature or changes to acid–base or electrolyte balance. For example, in liver failure, shunting of toxins from the gastrointestinal system that would otherwise be hepatically cleared and cerebral oedema co-exist. These processes contribute to a toxic-metabolic encephalopathy whose clinical manifestations can range from subtle drowsiness or confusion to fulminant coma [94]. Early detection of hepatic encephalopathy, especially when its manifestations are subtle (so-called minimal hepatic encephalopathy), can be difficult. However, intervention when mild encephalopathy is detected can mitigate more severe manifestations [95]. The Psychometric Hepatic Encephalopathy Score (PHES) is considered a gold standard for the diagnosis of minimal hepatic encephalopathy. Impairments in the PHES correlates with impaired performance on assessments of saccadic eye movements [61]. Specifically, Casanova-Ferrer et al. found significant linear correlations between worse PHES score and increased AS latency and error rate [61]. However, the authors primarily reported on the proportion of abnormal parameters from proprietary software analysis of the eye movements, many of which are derivative measurements. In any case, this is an early exploration of using saccadic tests as a biomarker in a specific disease context (that is, liver failure) where it can improve clinical detection and quantification of encephalopathy where there is a high pre-test probability of its existence. It also points towards analogous application in other causes of toxic-metabolic encephalopathy, which as a group is a prevalent disorder whose early detection is challenging but impacts clinical management.

### Cervical dystonia

Cervical dystonia is the most common focal dystonia which causes abnormal neck posturing, tremor and pain to varying degrees of severity [96]. While the pathogenesis is neurological and basal ganglia involvement has been established, work remains to describe the exact contributory mechanisms. A “head neural integrator” network has been proposed, in which network disruption results in malfunction in the constant process of maintaining head posture, thus resulting in cervical dystonia [97]. Beyond the basal ganglia, this network has been proposed to include the frontoparietal cortex, the brainstem and the cerebellum [98] – thus overlapping with areas involved in the cognitive control of eye movements [99] and, in particular, the DLPFC [100].

AS latency and error rate were both significantly increased in 31 cervical dystonia patients compared to healthy controls [62]. Furthermore, increased express saccades were seen in all tasks and more errors were seen in a no-go countermanding task, pointing towards inhibitory failure. While standard neuropsychological assessments were not performed on participants in this study, the authors cite relevant cognitive impairments described in cervical dystonia patients elsewhere [101]. However, further work is still required to clarify the nature of these correlations.

The application of saccadic eye testing in cervical dystonia furthers the known boundaries of an incompletely understood disease, where usual clinical assessment is unconcerned with cognition. While monitoring and management of this disease will undoubtedly remain focussed on its dystonic symptoms, saccadic analysis provides further avenues to clarify the neural networks involved in its pathogenesis.

## Expanding the role of ocular motor assessment in neurological disease

Overall, ocular motor studies across a gamut of disease states have been useful in clarifying brain pathology as well as refining theories about ocular motor control. They have allowed inferences to be made about the localisation or even the presence of brain pathology, and assessments taken across different time points of a disease process have revealed information about the persistence, progression or resolution of pathology.

Furthermore, in several neurological conditions explored here—such as mild traumatic brain injury, minor stroke and clinically isolated syndrome of MS—ocular motor studies have detected problems with saccadic parameters long after the patient has clinically recovered. In the case of mild cognitive impairment, disturbances in saccadic parameters can be found long before the manifestation of clinically definite dementia.

These findings substantiate the sensitivity of saccadic testing in peri-disease states to neuropsychological disturbances, pointing towards potential utility as a biomarker both in anticipation of future disease or in monitoring recovery or stability when the outcomes of routine clinical assessments have already returned to within normal limits.

In terms of clinical application, longitudinal studies of ocular motor function are currently lacking for all the diseases reviewed here, and this will be essential to establish saccadic parameters in a role as a biomarker. Furthermore, the cognitive effects of many medications used in treatment of neurological conditions may also influence saccadic parameters, which will require careful delineation especially if these results were to play a role in clinical trials.

Much work remains to be done with the standardisation of ocular motor testing paradigms as well as technical aspects of the equipment and saccadic analysis. For example, a large amount of heterogeneity is seen in the saccadic eye tests themselves, especially across the Alzheimer’s disease and schizophrenia literature, introducing significant challenges to systematic review or meta-analysis. A detailed understanding of the cognitive control of saccades is necessary to ensure that parametric nuances in testing paradigms do not introduce confounders—for example, gap conditions, switch costs as well as directionality of eye movements.

Antoniades et al. [102], Nij Bijvank et al. [103] and Demian et al. [104] have all sought to develop standardised saccadic test batteries with the corresponding normal standards. These aim to define testing and analysis protocols to facilitate comparison between studies and improve reproducibility of results. Agreement on testing approaches and standardisation of testing protocols will be necessary to produce research that can be compared longitudinally and across centres, which is currently limited by the heterogeneous methodology seen in the reviewed studies.

Video-oculography also produces large datasets that rapidly become time consuming to manually analyse. Automated analysis of eye movement tracings and saccadic parameters is necessary to summarise these large eye datasets generated in any single session for these tests to be practical. With automation algorithms, clearly defined methodology is required for selecting saccades, filtering artefacts and errors (such as blinks, intrusions, anticipation) and calculating derivative parameters. Li et al. [105] developed and defined such an algorithm for the automated analysis of video-oculographic recordings, which they applied to a patient population with neurodegenerative conditions. Their algorithm automatically selected saccades, blinks, fixations and pursuit movements and extracted their associated parameters. While they did not describe any specific quality control measures to assess the performance of their algorithm, the explicit documentation of the algorithm’s parameters provides an important basis for future interrogation and refinement of these techniques, especially in an area where these methodologies are yet to be standardised.

While the proliferation of eye tracking devices improves accessibility for researchers, the specific technical aspects of their hardware and software can introduce systematic errors especially if their configuration and analysis methodology is opaque or not immediately disclosed to the researcher. These opacities can be introduced by, for example, proprietary software or machine learning algorithms.

Several important technical factors will require standardisation. The speed of saccadic eye movements demands a higher sampling frequency than, for example, measuring pursuit movements, due to the precision required in determining the exact onset of a saccade in the millisecond range. Video hardware can sample eye movements at a rate ranging from 30 samples per second up to 1000 samples per second, but some degree of flexibility is possible here by compensating for the reduced accuracy of lower sampling rates by taking the average of a larger number of saccadic trials [106]. At the lower limits of sampling frequency, there are suggestions that a sampling rate of 120 Hz has advantages in saccadic detection over a sampling rate of 60 Hz, the latter of which especially struggles when assessing saccades with smaller amplitudes [107]. The specifics of tracking methodology in video-oculography are also evolving, with proliferation of appearance-based methods of tracking due to its lesser hardware requirements [108]. This approach relies on largely unenhanced images where the burden is placed on software to determine gaze cues, and while currently this offers overall lower spatial accuracy, rapid improvement in software techniques may change this in the near-future. Feature-based tracking, used especially in infrared video-oculography, allows determination of fixed ocular features including the pupil, corneal reflections and sometimes scleral landmarks within a calibrated geometry and determines the gaze direction with much higher spatial accuracy. However, depending on the testing paradigms required in any particular cognitive application, spatial accuracy may not be an important parameter if directional error (in the AS task) and latency are enough to appreciate cognitive disruption, which is the case in most of the neurological diseases reviewed here. Finally, the display screen itself, which encompasses details such as contrast and brightness, will affect the saliency of targets which can subsequently impact saccadic performance [109, 110]. These are all factors that can differentially affect measurements in different cohorts, where standardisation will be required to facilitate between-study comparisons.

Overall, the saccadic testing paradigms to assess cognitive function, based on an established neuroanatomical model of ocular motor control, along with the consistent demonstration of its impairment in various neurological diseases, establishes precedent to pursue ocular motor assessment as a biomarker in many neurological conditions. The widespread presence of cognitive dysfunction in neurological disease, in general, indicates that disease impacts will likely be demonstrable with ocular motor assessments of saccadic eye movement. There is potential for detecting saccadic eye movement abnormalities to help support diagnostic workup, prognosticate disease course or monitor for recovery or relapse. Its strengths lie in sensitivity to cognitive dysfunction that will extend routine clinical tests through practical and precise measurement of the ocular motor system.

## Data Availability

Not applicable.

## Abbreviations

ACC: Anterior cingulate cortex
AD: Alzheimer's dementia
ADL: Activities of daily living
AE: Autoimmune encephalitis
AS: Antisaccade
BG: Basal Ganglia
C5: Complement component 5
CIS: Clinically isolated syndrome
CNS: Central nervous system
CSF: Cerebrospinal fluid
CVLT: California Verbal Learning Test
DLPFC: Dorsal lateral prefrontal cortex
DRE: Drug-resistant epilepsy
EDSS: Expanded disability status scale
EEG: Electroencephalogram
FEF: Frontal eye fields
FLE: Frontal lobe epilepsy
GP: Globus pallidus
IPS: Interparietal sulcus
IQ: Intelligence quotient
LGN: Lateral geniculate nucleus
MCI: Mild cognitive impairment
MMSE: Mini-mental status examination
MRI: Magnetic resonance imaging
mRS: Modified Rankin score
MS: Multiple sclerosis
NART: National Adult Reading Test
NIHSS: National Institute of Health Stroke Scale
OM: Ocular motor
PASAT: Paced Auditory Serial Addition Test
PEF: Parietal eye field
PET: Positron emission tomography
PFC: Prefrontal cortex
PPC: Posterior parietal cortex
PPRF: Paramedian pontine reticular formation
PS: Prosaccade
RCT: Randomised controlled trial
RNS: Repetitive nerve stimulation
SC: Superior colliculus
SD: Standard deviation
SDMT: Symbol Digit Modalities Test
SEF: Supplementary eye field
SN: Substantia nigra
SRC: Sports-related concussion
TLE: Temporal lobe epilepsy
WAIS: Wechsler Adult Intelligence Scale

## References

[1] Coe BC, Munoz DP (2017) Mechanisms of saccade suppression revealed in the anti-saccade task. Phil Trans R Soc Lond B Biol Sci 372:20160192. 10.1098/rstb.2016.0192 28242726 10.1098/rstb.2016.0192 PMC5332851

[2] Hutton SB (2008) Cognitive control of saccadic eye movements. Brain Cogn 68:327–340. 10.1016/j.bandc.2008.08.021 19028265 10.1016/j.bandc.2008.08.021

[3] Leigh RJ, Zee DS (2015) The neurology of eye movements, 5th edn. Oxford University Press, Oxford

[4] Rayner K (2009) The 35th Sir Frederick Bartlett lecture: eye movements and attention in reading, scene perception, and visual search. Q J Exp Psychol 62:1457–1506. 10.1080/17470210902816461 10.1080/17470210902816461 19449261

[5] Scudder C, Kaneko C, Fuchs A (2002) The brainstem burst generator for saccadic eye movements. Exp Brain Res 142:439–462. 10.1007/s00221-001-0912-9 11845241 10.1007/s00221-001-0912-9

[6] Carpenter RHS (1994) Frontal cortex: choosing where to look. Curr Biol 4:341–343. 10.1016/S0960-9822(00)00074-9 7922343 10.1016/s0960-9822(00)00074-9

[7] Buttner-Ennever JA, Horn AKE, Henn V, Cohen B (1999) Projections from the superior colliculus motor map to omnipause neurons in monkey. J Comp Neurol 413:55–67. 10.1002/(SICI)1096-9861(19991011)413 10464369 10.1002/(sici)1096-9861(19991011)413:1<55::aid-cne3>3.0.co;2-k

[8] Jayaraman A, Batton RR, Carpenter MB (1977) Nigrotectal projections in the monkey: an autoradiographic study. Brain Res 135:147–152. 10.1016/0006-8993(77)91058-7 410480 10.1016/0006-8993(77)91058-7

[9] Shires J, Joshi S, Basso MA (2010) Shedding new light on the role of the basal ganglia-superior colliculus pathway in eye movements. Curr Opin Neurobiol 20:717–725. 10.1016/j.conb.2010.08.008 20829033 10.1016/j.conb.2010.08.008 PMC3008502

[10] Hikosaka O, Takikawa Y, Kawagoe R (2000) Role of the basal ganglia in the control of purposive saccadic eye movements. Physiol Rev 80:953–978. 10.1152/physrev.2000.80.3.953 10893428 10.1152/physrev.2000.80.3.953

[11] Pierrot-Deseilligny C, Müri RM, Ploner CJ et al (2003) Cortical control of ocular saccades in humans: a model for motricity. In: Swaab D, Waxman S (eds) Progress in brain research. Elsevier, Amsterdam, pp 3–17 10.1016/S0079-6123(03)42003-7 12693251

[12] Schiller PH, True SD, Conway JL (1980) Deficits in eye movements following frontal eye-field and superior colliculus ablations. J Neurophysiol 44:1175–1189. 10.1152/jn.1980.44.6.1175 6778974 10.1152/jn.1980.44.6.1175

[13] Segraves MA (1992) Activity of monkey frontal eye field neurons projecting to oculomotor regions of the pons. J Neurophysiol 68:1967–1985. 10.1152/jn.1992.68.6.1967 1491252 10.1152/jn.1992.68.6.1967

[14] Barash S, Bracewell RM, Fogassi L et al (1991) Saccade-related activity in the lateral intraparietal area. I. Temporal properties; comparison with area 7a. J Neurophysiol 66:1095–1108. 10.1152/jn.1991.66.3.1095 1753276 10.1152/jn.1991.66.3.1095

[15] Pierrot-Deseilligny C, Milea D, Muri RM (2004) Eye movement control by the cerebral cortex. Curr Opin Neurol 17:17–25. 10.1097/00019052-200402000-00005 15090873 10.1097/00019052-200402000-00005

[16] Krauzlis RJ (2005) The control of voluntary eye movements: new perspectives. Neuroscientist 11:124–137. 10.1177/1073858404271196 15746381 10.1177/1073858404271196

[17] Robinson FR, Fuchs AF (2001) The role of the cerebellum in voluntary eye movements. Annu Rev Neurosci 24:981–1004. 10.1146/annurev.neuro.24.1.981 11520925 10.1146/annurev.neuro.24.1.981

[18] Hansen DW, Ji Q (2010) In the eye of the beholder: a survey of models for eyes and gaze. IEEE Trans Pattern Anal Mach Intell 32:478–500. 10.1109/TPAMI.2009.30 20075473 10.1109/TPAMI.2009.30

[19] Hunt AR, Kingstone A (2003) Covert and overt voluntary attention: linked or independent? Cogn Brain Res 18:102–105. 10.1016/j.cogbrainres.2003.08.006 10.1016/j.cogbrainres.2003.08.006 14659502

[20] Rizzolatti G, Riggio L, Dascola I, Umiltá C (1987) Reorienting attention across the horizontal and vertical meridians: evidence in favor of a premotor theory of attention. Neuropsychologia 25:31–40. 10.1016/0028-3932(87)90041-8 3574648 10.1016/0028-3932(87)90041-8

[21] Beauchamp MS, Petit L, Ellmore TM et al (2001) A parametric fMRI study of overt and covert shifts of visuospatial attention. Neuroimage 14:310–321. 10.1006/nimg.2001.0788 11467905 10.1006/nimg.2001.0788

[22] Fischer B, Weber H (1992) Characteristics of ?anti? saccades in man. Exp Brain Res. 10.1007/BF00228257 1623983 10.1007/BF00228257

[23] Fischer B, Weber H (1993) Express saccades and visual attention. Behav Brain Sci 16:553–567. 10.1017/S0140525X00031575

[24] Everling S, Paré M, Dorris MC, Munoz DP (1998) Comparison of the discharge characteristics of brain stem omnipause neurons and superior colliculus fixation neurons in monkey: implications for control of fixation and saccade behavior. J Neurophysiol 79:511–528. 10.1152/jn.1998.79.2.511 9463418 10.1152/jn.1998.79.2.511

[25] Kingstone A, Klein RM (1993) Visual offsets facilitate saccadic latency: does predisengagement of visuospatial attention mediate this gap effect? J Exp Psychol Hum Percept Perform 19:1251–1265. 10.1037/0096-1523.19.6.1251 8294890 10.1037//0096-1523.19.6.1251

[26] Carpenter RHS (2001) Express saccades: is bimodality a result of the order of stimulus presentation? Vis Res 41:1145–1151. 10.1016/S0042-6989(01)00007-4 11292505 10.1016/s0042-6989(01)00007-4

[27] Dorris MC, Paré M, Munoz DP (1997) Neuronal activity in monkey superior colliculus related to the initiation of saccadic eye movements. J Neurosci 17:8566–8579. 10.1523/JNEUROSCI.17-21-08566.1997 9334428 10.1523/JNEUROSCI.17-21-08566.1997 PMC6573744

[28] Edelman JA, Kristjánsson Á, Nakayama K (2007) The influence of object-relative visuomotor set on express saccades. J Vis 7:12. 10.1167/7.6.12 17685795 10.1167/7.6.12

[29] McDowell JE, Dyckman KA, Austin BP, Clementz BA (2008) Neurophysiology and neuroanatomy of reflexive and volitional saccades: evidence from studies of humans. Brain Cogn 68:255–270. 10.1016/j.bandc.2008.08.016 18835656 10.1016/j.bandc.2008.08.016 PMC2614688

[30] Mansouri FA, Buckley MJ, Tanaka K (2007) Mnemonic function of the dorsolateral prefrontal cortex in conflict-induced behavioral adjustment. Science 318:987–990. 10.1126/science.1146384 17962523 10.1126/science.1146384

[31] Pierrot-Deseilligny C, Müri RM, Ploner CJ et al (2003) Decisional role of the dorsolateral prefrontal cortex in ocular motor behaviour. Brain 126:1460–1473. 10.1093/brain/awg148 12764065 10.1093/brain/awg148

[32] Hutton SB, Ettinger U (2006) The antisaccade task as a research tool in psychopathology: a critical review. Psychophysiology 43:302–313. 10.1111/j.1469-8986.2006.00403.x 16805870 10.1111/j.1469-8986.2006.00403.x

[33] Reuter B, Kaufmann C, Bender J et al (2010) Distinct neural correlates for volitional generation and inhibition of saccades. J Cogn Neurosci 22:728–738. 10.1162/jocn.2009.21235 19366286 10.1162/jocn.2009.21235

[34] Connolly JD, Goodale MA, Desouza JFX et al (2000) A comparison of frontoparietal fMRI activation during anti-saccades and anti-pointing. J Neurophysiol 84:1645–1655 10980034 10.1152/jn.2000.84.3.1645

[35] Doricchi F, Perani D, Incoccia C et al (1997) Neural control of fast-regular saccades and antisaccades: an investigation using positron emission tomography. Exp Brain Res 116:50–62. 10.1007/PL00005744 9305814 10.1007/pl00005744

[36] Weiler J, Heath M (2012) Task-switching in oculomotor control: unidirectional switch-cost when alternating between pro- and antisaccades. Neurosci Lett 530:150–154. 10.1016/j.neulet.2012.10.007 23063688 10.1016/j.neulet.2012.10.007

[37] Herd SA, O’Reilly RC, Hazy TE et al (2014) A neural network model of individual differences in task switching abilities. Neuropsychologia 62:375–389. 10.1016/j.neuropsychologia.2014.04.014 24791709 10.1016/j.neuropsychologia.2014.04.014 PMC4167201

[38] Pierrot-Deseilligny C, Rivaud S, Gaymard B, Agid Y (1991) Cortical control of memory-guided saccades in man. Exp Brain Res. 10.1007/BF00229839 2026201 10.1007/BF00229839

[39] Krappmann P, Everling S, Flohr H (1998) Accuracy of visually and memory-guided antisaccades in man. Vis Res 38:2979–2985. 10.1016/s0042-6989(98)00101-1 9797993 10.1016/s0042-6989(98)00101-1

[40] Ploner CJ, Gaymard B, Rivaud S et al (1998) Temporal limits of spatial working memory in humans. Eur J Neurosci 10:794–797. 10.1046/j.1460-9568.1998.00101.x 9749746 10.1046/j.1460-9568.1998.00101.x

[41] Jeter CB, Patel SS, Sereno AB (2011) Novel n-back spatial working memory task using eye movement response. Behav Res 43:879–887. 10.3758/s13428-011-0093-9 10.3758/s13428-011-0093-9 PMC4821187 21487898

[42] Molitor RJ, Ko PC, Ally BA (2015) Eye movements in Alzheimer’s disease. JAD 44:1–12. 10.3233/JAD-141173 25182738 10.3233/JAD-141173 PMC5332166

[43] Opwonya J, Doan DNT, Kim SG et al (2022) Saccadic eye movement in mild cognitive impairment and Alzheimer’s disease: a systematic review and meta-analysis. Neuropsychol Rev 32:193–227. 10.1007/s11065-021-09495-3 33959887 10.1007/s11065-021-09495-3 PMC9090874

[44] Antoniades CA, Demeyere N, Kennard C et al (2015) Antisaccades and executive dysfunction in early drug-naive Parkinson’s disease: the discovery study. Mov Disord 30:843–847. 10.1002/mds.26134 25600361 10.1002/mds.26134

[45] Chen Y-F, Chen T, Tsai T-T (1999) Analysis of volition latency on antisaccadic eye movements. Med Eng Phys 21:555–562. 10.1016/S1350-4533(99)00082-X 10672789 10.1016/s1350-4533(99)00082-x

[46] Chambers JM, Prescott TJ (2010) Response times for visually guided saccades in persons with Parkinson’s disease: a meta-analytic review. Neuropsychologia 48:887–899. 10.1016/j.neuropsychologia.2009.11.006 19913042 10.1016/j.neuropsychologia.2009.11.006

[47] Chan F, Armstrong IT, Pari G et al (2005) Deficits in saccadic eye-movement control in Parkinson’s disease. Neuropsychologia 43:784–796. 10.1016/j.neuropsychologia.2004.06.026 15721191 10.1016/j.neuropsychologia.2004.06.026

[48] Fielding J, Kilpatrick T, Millist L et al (2012) Longitudinal assessment of antisaccades in patients with multiple sclerosis. PLoS ONE 7:e30475. 10.1371/journal.pone.0030475 22319570 10.1371/journal.pone.0030475 PMC3271102

[49] Clough M, Millist L, Lizak N et al (2015) Ocular motor measures of cognitive dysfunction in multiple sclerosis I: inhibitory control. J Neurol 262:1130–1137. 10.1007/s00415-015-7645-3 25851743 10.1007/s00415-015-7645-3

[50] Clough M, Mitchell L, Millist L et al (2015) Ocular motor measures of cognitive dysfunction in multiple sclerosis II: working memory. J Neurol 262:1138–1147. 10.1007/s00415-015-7644-4 25851742 10.1007/s00415-015-7644-4

[51] Dong W, Yan B, Johnson BP et al (2013) Ischaemic stroke: the ocular motor system as a sensitive marker for motor and cognitive recovery. J Neurol Neurosurg Psychiatry 84:337–341. 10.1136/jnnp-2012-303926 23223333 10.1136/jnnp-2012-303926 PMC3582066

[52] Clough M, Mutimer S, Wright DK et al (2018) Oculomotor cognitive control abnormalities in Australian rules football players with a history of concussion. J Neurotrauma 35:730–738. 10.1089/neu.2017.5204 29228862 10.1089/neu.2017.5204

[53] Omalu BI, DeKosky ST, Hamilton RL et al (2006) Chronic traumatic encephalopathy in a national football league player: part II. Neurosurgery 59:1086–1092. 10.1227/01.NEU.0000245601.69451.27 17143242 10.1227/01.NEU.0000245601.69451.27

[54] Kraus MF, Little DM, Donnell AJ et al (2007) Oculomotor function in chronic traumatic brain injury. Cogn Behav Neurol 20:170–178. 10.1097/WNN.0b013e318142badb 17846516 10.1097/WNN.0b013e318142badb

[55] Ting WK-C, Schweizer TA, Topolovec-Vranic J, Cusimano MD (2016) Antisaccadic eye movements are correlated with corpus callosum white matter mean diffusivity, stroop performance, and symptom burden in mild traumatic brain injury and concussion. Front Neurol. 10.3389/fneur.2015.00271 26834693 10.3389/fneur.2015.00271 PMC4716139

[56] Brooks JS, Dickey JP (2024) Effect of repetitive head impacts on saccade performance in Canadian University Football players. Clin J Sport Med. 10.1097/JSM.0000000000001202 38150378 10.1097/JSM.0000000000001202 PMC11042529

[57] Au Yong HM, Clough M, Perucca P et al (2023) Ocular motility as a measure of cerebral dysfunction in adults with focal epilepsy. Epilepsy Behav 141:109140. 10.1016/j.yebeh.2023.109140 36812874 10.1016/j.yebeh.2023.109140

[58] Gooding DC, Basso MA (2008) The tell-tale tasks: a review of saccadic research in psychiatric patient populations. Brain Cogn 68:371–390. 10.1016/j.bandc.2008.08.024 18950927 10.1016/j.bandc.2008.08.024 PMC2755089

[59] Hutton SB, Huddy V, Barnes TRE et al (2004) The relationship between antisaccades, smooth pursuit, and executive dysfunction in first-episode schizophrenia. Biol Psychiatry 56:553–559. 10.1016/j.biopsych.2004.07.002 15476684 10.1016/j.biopsych.2004.07.002

[60] Ettinger U, Kumari V, Chitnis XA et al (2004) Volumetric neural correlates of antisaccade eye movements in first-episode psychosis. AJP 161:1918–1921. 10.1176/ajp.161.10.1918 10.1176/ajp.161.10.1918 15465994

[61] Casanova-Ferrer F, García-Cena CE, Gallego J-J et al (2022) Minimal hepatic encephalopathy is associated to alterations in eye movements. Sci Rep 12:16837. 10.1038/s41598-022-21230-3 36207472 10.1038/s41598-022-21230-3 PMC9547018

[62] Carbone F, Ellmerer P, Ritter M et al (2021) Impaired inhibitory control of saccadic eye movements in cervical dystonia: an eye-tracking study. Mov Disord 36:1246–1250. 10.1002/mds.28486 33416199 10.1002/mds.28486 PMC8247854

[63] Diefendorf AR, Dodge R (1908) An experimental study of the ocular reactions of the insane from photographic records. Brain 31:451–489. 10.1093/brain/31.3.451

[64] Townsend L, Pillinger T, Selvaggi P et al (2023) Brain glucose metabolism in schizophrenia: a systematic review and meta-analysis of 18FDG-PET studies in schizophrenia. Psychol Med 53:4880–4897. 10.1017/S003329172200174X 35730361 10.1017/S003329172200174X PMC10476075

[65] Fukushima J, Fukushima K, Chiba T et al (1988) Disturbances of voluntary control of saccadic eye movements in schizophrenic patients. Biol Psychiatry 23:670–677. 10.1016/0006-3223(88)90050-9 3370264 10.1016/0006-3223(88)90050-9

[66] Hutton SB, Crawford TJ, Puri BK et al (1998) Smooth pursuit and saccadic abnormalities in first-episode schizophrenia. Psychol Med 28:685–692. 10.1017/S0033291798006722 9626724 10.1017/s0033291798006722

[67] Zhu J, Zhou L, Zhou Y et al (2024) Diagnosis of schizophrenia by integrated saccade scores and associations with psychiatric symptoms, and functioning. Medicine 103:e39935. 10.1097/MD.0000000000039935 39465854 10.1097/MD.0000000000039935 PMC11479490

[68] Sweeney JA, Strojwas MH, Mann JJ, Thase ME (1998) Prefrontal and cerebellar abnormalities in major depression: evidence from oculomotor studies. Biol Psychiatry 43:584–594. 10.1016/S0006-3223(97)00485-X 9564443 10.1016/s0006-3223(97)00485-x

[69] Winograd-Gurvich C, Georgiou-Karistianis N, Fitzgerald PB et al (2006) Ocular motor differences between melancholic and non-melancholic depression. J Affect Disord 93:193–203. 10.1016/j.jad.2006.03.018 16678910 10.1016/j.jad.2006.03.018

[70] Derakshan N, Ansari TL, Hansard M et al (2009) Anxiety, inhibition, efficiency, and effectiveness: an investigation using the antisaccade task. Exp Psychol 56:48–55. 10.1027/1618-3169.56.1.48 19261578 10.1027/1618-3169.56.1.48

[71] Hyman BT, Phelps CH, Beach TG et al (2012) National institute on aging-Alzheimer’s association guidelines for the neuropathologic assessment of Alzheimer’s disease. Alzheimers Dement 8:1–13. 10.1016/j.jalz.2011.10.007 22265587 10.1016/j.jalz.2011.10.007 PMC3266529

[72] Silverman DHS, Small GW, Chang CY et al (2001) Positron emission tomography in evaluation of dementia: regional brain metabolism and long-term outcome. JAMA 286:2120. 10.1001/jama.286.17.2120 11694153 10.1001/jama.286.17.2120

[73] Wolf A, Tripanpitak K, Umeda S, Otake-Matsuura M (2023) Eye-tracking paradigms for the assessment of mild cognitive impairment: a systematic review. Front Psychol 14:1197567. 10.3389/fpsyg.2023.1197567 37546488 10.3389/fpsyg.2023.1197567 PMC10399700

[74] Yang Q, Wang T, Su N et al (2013) Specific saccade deficits in patients with Alzheimer’s disease at mild to moderate stage and in patients with amnestic mild cognitive impairment. Age 35:1287–1298. 10.1007/s11357-012-9420-z 22576337 10.1007/s11357-012-9420-z PMC3705110

[75] Bondi MW, Edmonds EC, Salmon DP (2017) Alzheimer’s disease: past, present, and future. J Int Neuropsychol Soc 23:818–831. 10.1017/S135561771700100X 29198280 10.1017/S135561771700100X PMC5830188

[76] Rane D, Dash DP, Dutt A et al (2023) Distinctive visual tasks for characterizing mild cognitive impairment and dementia using oculomotor behavior. Front Aging Neurosci 15:1125651. 10.3389/fnagi.2023.1125651 37547742 10.3389/fnagi.2023.1125651 PMC10397802

[77] Curtis CE, D’Esposito M (2003) Persistent activity in the prefrontal cortex during working memory. Trends Cogn Sci 7:415–423. 10.1016/s1364-6613(03)00197-9 12963473 10.1016/s1364-6613(03)00197-9

[78] Holden JG, Cosnard A, Laurens B et al (2018) Prodromal Alzheimer’s disease demonstrates increased errors at a simple and automated anti-saccade task. JAD 65:1209–1223. 10.3233/JAD-180082 30149445 10.3233/JAD-180082

[79] Chehrehnegar N, Shati M, Esmaeili M, Foroughan M (2022) Executive function deficits in mild cognitive impairment: evidence from saccade tasks. Aging Ment Health 26:1001–1009. 10.1080/13607863.2021.1913471 33928806 10.1080/13607863.2021.1913471

[80] Jankovic J, Tan EK (2020) Parkinson’s disease: etiopathogenesis and treatment. J Neurol Neurosurg Psychiatry 91:795–808. 10.1136/jnnp-2019-322338 32576618 10.1136/jnnp-2019-322338

[81] Roll A, Wierzbicka MM, Wolf W (1996) The ?gap paradigm? leads to express-like saccadic reaction times in Parkinson’s disease. Exp Brain Res. 10.1007/BF00229562 8891643 10.1007/BF00229562

[82] Henik A, Singh J, Beckley DJ, Rafal RD (1993) Disinhibition of automatic word reading in Parkinson’s disease. Cortex 29:589–599. 10.1016/S0010-9452(13)80283-3 8124936 10.1016/s0010-9452(13)80283-3

[83] Ekman U, Eriksson J, Forsgren L et al (2012) Functional brain activity and presynaptic dopamine uptake in patients with Parkinson’s disease and mild cognitive impairment: a cross-sectional study. Lancet Neurol 11:679–687. 10.1016/S1474-4422(12)70138-2 22742929 10.1016/S1474-4422(12)70138-2

[84] Kövari E, Gold G, Herrmann FR et al (2003) Lewy body densities in the entorhinal and anterior cingulate cortex predict cognitive deficits in Parkinson’s disease. Acta Neuropathol 106:83–88. 10.1007/s00401-003-0705-2 12687392 10.1007/s00401-003-0705-2

[85] Stadelmann C (2011) Multiple sclerosis as a neurodegenerative disease: pathology, mechanisms and therapeutic implications. Curr Opin Neurol 24:224–229. 10.1097/WCO.0b013e328346056f 21455066 10.1097/WCO.0b013e328346056f

[86] Fielding J, Kilpatrick T, Millist L, White O (2009) Multiple sclerosis: cognition and saccadic eye movements. J Neurol Sci 277:32–36. 10.1016/j.jns.2008.10.001 18977003 10.1016/j.jns.2008.10.001

[87] Lizak N, Clough M, Millist L et al (2016) Impairment of smooth pursuit as a marker of early multiple sclerosis. Front Neurol. 10.3389/fneur.2016.00206 27917151 10.3389/fneur.2016.00206 PMC5116770

[88] Smith EE (2017) Clinical presentations and epidemiology of vascular dementia. Clin Sci Lond 131:1059–1068. 10.1042/CS20160607 28515342 10.1042/CS20160607

[89] Rivaud S, Muri RM, Gaymard B et al (1994) Eye movement disorders after frontal eye field lesions in humans. Exp Brain Res. 10.1007/BF00232443 7895787 10.1007/BF00232443

[90] Povlishock JT, Katz DI (2005) Update of neuropathology and neurological recovery after traumatic brain injury. J Head Trauma Rehabil 20:76–94. 10.1097/00001199-200501000-00008 15668572 10.1097/00001199-200501000-00008

[91] Fisher RS, Acevedo C, Arzimanoglou A et al (2014) Ilae official report: a practical clinical definition of epilepsy. Epilepsia 55:475–482. 10.1111/epi.12550 24730690 10.1111/epi.12550

[92] Aldenkamp AP (2006) Cognitive impairment in epilepsy: state of affairs and clinical relevance. Seizure 15:219–220. 10.1016/j.seizure.2006.02.010

[93] Elger CE, Helmstaedter C, Kurthen M (2004) Chronic epilepsy and cognition. Lancet Neurol 3:663–672. 10.1016/S1474-4422(04)00906-8 15488459 10.1016/S1474-4422(04)00906-8

[94] Fraser CL, Arieff AI (1985) Hepatic encephalopathy. N Engl J Med 313:865–873. 10.1056/NEJM198510033131406 2993889 10.1056/NEJM198510033131406

[95] Patidar KR, Bajaj JS (2015) Covert and overt hepatic encephalopathy: diagnosis and management. Clin Gastroenterol Hepatol 13:2048–2061. 10.1016/j.cgh.2015.06.039 26164219 10.1016/j.cgh.2015.06.039 PMC4618040

[96] LaHue SC, Albers K, Goldman S et al (2020) Cervical dystonia incidence and diagnostic delay in a multiethnic population. Mov Disord 35:450–456. 10.1002/mds.27927 31774238 10.1002/mds.27927 PMC10683845

[97] Klier EM, Wang H, Constantin AG, Crawford JD (2002) Midbrain control of three-dimensional head orientation. Science 295:1314–1316. 10.1126/science.1067300 11847347 10.1126/science.1067300

[98] Giannì C, Pasqua G, Ferrazzano G et al (2022) Focal dystonia: functional connectivity changes in cerebellar-basal ganglia-cortical circuit and preserved global functional architecture. Neurology 98:e1499–e1509. 10.1212/WNL.0000000000200022 35169015 10.1212/WNL.0000000000200022

[99] Shaikh AG, Zee DS, Crawford JD, Jinnah HA (2016) Cervical dystonia: a neural integrator disorder. Brain 139:2590–2599. 10.1093/brain/aww141 27324878 10.1093/brain/aww141 PMC5840887

[100] Prell T, Peschel T, Köhler B et al (2013) Structural brain abnormalities in cervical dystonia. BMC Neurosci 14:123. 10.1186/1471-2202-14-123 24131497 10.1186/1471-2202-14-123 PMC3852757

[101] Romano R, Bertolino A, Gigante A et al (2014) Impaired cognitive functions in adult-onset primary cranial cervical dystonia. Parkinsonism Relat Disord 20:162–165. 10.1016/j.parkreldis.2013.10.008 24161376 10.1016/j.parkreldis.2013.10.008

[102] Antoniades C, Ettinger U, Gaymard B et al (2013) An internationally standardised antisaccade protocol. Vis Res 84:1–5. 10.1016/j.visres.2013.02.007 23474300 10.1016/j.visres.2013.02.007

[103] Nij Bijvank JA, Petzold A, Balk LJ et al (2018) A standardized protocol for quantification of saccadic eye movements: DEMoNS. PLoS ONE 13:e0200695. 10.1371/journal.pone.0200695 30011322 10.1371/journal.pone.0200695 PMC6047815

[104] Demian D, Petrak M, Zielinski G et al (2023) Clinical saccadometry: establishing evaluative standards using a simplified video oculography protocol in the adult population. J Am Acad Audiol. 10.1055/s-0043-1772582 37989200 10.1055/s-0043-1772582 PMC11479743

[105] Li D, Butala AA, Moro-Velazquez L et al (2024) Automating the analysis of eye movement for different neurodegenerative disorders. Comput Biol Med 170:107951. 10.1016/j.compbiomed.2024.107951 38219646 10.1016/j.compbiomed.2024.107951

[106] Andersson R, Nyström M, Holmqvist K (2010) Sampling frequency and eye-tracking measures: how speed affects durations, latencies, and more. JEMR. 10.16910/jemr.3.3.6

[107] Leube A, Rifai K, Rifai K (2017) Sampling rate influences saccade detection in mobile eye tracking of a reading task. J Eye Mov Res. 10.16910/jemr.10.3.3 33828659 10.16910/jemr.10.3.3 PMC7141092

[108] Larrazabal AJ, García Cena CE, Martínez CE (2019) Video-oculography eye tracking towards clinical applications: a review. Comput Biol Med 108:57–66. 10.1016/j.compbiomed.2019.03.025 31003180 10.1016/j.compbiomed.2019.03.025

[109] Henderson JM, Nuthmann A, Luke SG (2013) Eye movement control during scene viewing: immediate effects of scene luminance on fixation durations. J Exp Psychol Hum Percept Perform 39:318–322. 10.1037/a0031224 23276111 10.1037/a0031224

[110] Näsänen R, Ojanpää H, Kojo I (2001) Effect of stimulus contrast on performance and eye movements in visual search. Vision Res 41:1817–1824. 10.1016/S0042-6989(01)00056-6 11369045 10.1016/s0042-6989(01)00056-6

